# Hyperuricemia and acute kidney injury secondary to spontaneous tumor lysis syndrome in low risk myelodysplastic syndrome

**DOI:** 10.1186/1471-2369-15-164

**Published:** 2014-10-11

**Authors:** Yunlin Feng, Tao Jiang, Li Wang

**Affiliations:** Division of Nephrology, Sichuan Academy of Medical Sciences and Sichuan Provincial People’s Hospital, Chengdu, 610072 China; Division of Hemotology, Sichuan Academy of Medical Sciences and Sichuan Provincial People’s Hospital, Chengdu, 610072 China

**Keywords:** Acute kidney injury, Hyperuricemia, Myelodysplastic syndrome, Spontaneous tumor lysis syndrome

## Abstract

**Background:**

This is a rare instance of acute kidney injury caused by hyperuricemia due to spontaneous tumor lysis syndrome and also the first case of spontaneous tumor lysis syndrome reported in association with myelodysplastic syndrome.

**Case presentation:**

A 53-year-old man presented with abrupt oliguria. Laboratory findings on admission included hyperuricemia, hyperphosphatemia, hypocalcemia, metabolic acidosis and rapidly rising serum creatinine, which were consistent with acute tumor lysis syndrome in the absence of precipitating chemotherapy or radiotherapy. After hemodialysis and oral uric acid lowering therapy, serum uric acid levels returned to normal range and renal function rapidly recovered. The patient was diagnosed as myelodysplastic syndrome eleven months later.

**Conclusions:**

Occult malignancy including solid tumors and hematological malignancies should be carefully evaluated in the case of unexplainable acute kidney injury with hyperuricemia. Aggressive investigations should be thoroughly considered and repeated in this population.

## Background

Acute tumor lysis syndrome (TLS) is a metabolic disorder manifesting as abrupt occurrence of acute kidney injury (AKI), metabolic acidosis and electrolyte disturbances which include hyperuricemia, hyperphosphatemia, hyperkalemia and hypocalcemia [1]. TLS most commonly results from treatment of malignancies, especially high turnover rate hematologic malignancies. Spontaneous tumor lysis syndrome (STLS) before chemotherapy is a rare event which has been mostly reported in Burkitt lymphoma and non T-cell acute lymphoblastic leukemia [2]. Here we report a rare case of STLS in myelodysplastic syndrome (MDS) which presented with marked hyperuricemia and AKI months before the diagnosis of MDS.

## Case presentation

A 53-year-old man presented to the Nephrology department with generalized malaise and decreased urine volume for 5 days. He denied nausea, vomiting or fever. The medical history included hypertension for 10 years and type 2 diabetes mellitus for 8 years, both of which had been well controlled. One week before the admission, he underwent laparoscopic cholecystectomy (LC) for right upper quadrant abdominal pain, which had been diagnosed as cholelithiasis and cholecystitis. The laboratory data pre- and post-op are summarized in Table 1.

**Table 1:** **Laboratory investigation results**

Date	WBCs	PLT	HGB	SCr	UA	CO_2_	K	cCa	P
	(/mm^3^)	(/mm^3^)	(g/dL)	(mg/dL)	(mg/dL)	(mEq/L)	(mEq/L)	(mEq/L)	(mEq/L)
Before LC	2130	218000	7.9	1.3	14.6	22.6	3.88	2.15	1.34
5 days before admission/after LC	3750	40000	6.1	1.4	15.3	22.1	3.12	2.02	1.14
1 day before admission	4500	134000	5.8	9.5	37.7	16.3	3.54	1.98	2.71
Admission	3970	146000	5.2	11.7	39.8	16.7	3.59	2.11	3.06
1 day after admission	4120	130000	6.2	10.2	31.4	17.7	3.74	2.20	2.52
3 days after admission	2310	165000	7.0	8.5	24.2	22.7	3.69	2.43	1.48
Discharge	5200	156000	7.7	1.1	6.2	23.6	4.42	2.31	1.56

Abbreviations: *LC* laparoscopic cholecystectomy, *WBCs* white blood cells, *PLT* platelets, *HGB* hemoglobin, *SCr* serum creatinine, *UA* uric acid, *CO*_*2*_ bicarbonate, *K* potassium, *cCa* corrected calcium, *P* phosphate.

At the current admission, physical examination showed a chronically ill-appearing man in no acute distress, with stable vital signs. The patient was awake, alert and oriented. There was no increase in jugular venous pressure. Pulmonary and cardiac examinations were unremarkable. Abdominal examination showed well healed small scars left by the LC. Mild bilateral pretibial pitting edema was present in both lower limbs. Neurological examination was unremarkable.

The results of an electrocardiogram and a chest/abdominal CT were unremarkable. An ultrasound showed relatively enlarged kidney size (right, 11.1 × 5.1 cm; left, 11.8 × 5.9 cm). Further investigations including complete blood count (CBC), serum creatinine (SCr), urea nitrogen (BUN), electrolytes, liver function, serum uric acid (UA), electrolytes and serum biomarkers. Laboratory results summarized in Table 1 indicated elevated SCr, hyperuricemia, hyperphosphatemia, hypocalcemia as well as severe anemia and a slightly decreased WBC count. The remaining of lab tests, including serum tumor biomarkers were normal. The patient’s clinical manifestations of AKI and electrolyte disturbances were consistent with acute TLS.

**Figure 1:**
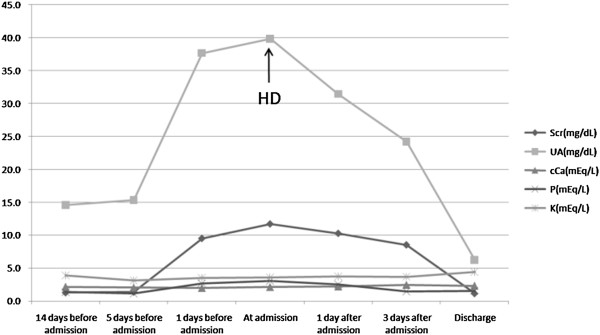
**Serial biochemical study of the patient.** Note: SCr, in mg/dL, where 1 mg/dL = 88.4 μEq/L; UA, in mg/dL, where 1 mg/dL = 59.48 μEq/L. Abbreviation: HD, hemodialysis.

After admission, the patient was transfused and intermittently hemodialysed, after which SCr and uric acid (UA) decreased gradually, with diminution of edema. Two weeks later, SCr had almost returned to normal and dialysis was stopped. Changes of serum biochemical parameters are shown in Figure 1. Since the anemia continued and there were no signs of solid tumors, we highly suspected hematological malignancies and performed several bone marrow examinations. Bone marrow smears from the first two showed no signs of malignancies. There was mild dyshaematopoiesis on the third examination but was not possible to make a definite diagnosis. The biopsy was normal and the molecular analysis returned negative.

The patient visited the clinic every 2-3 months after discharge. Investigations showed HGB was fluctuating in the range of 7.5-9.0 g/dL while SCr and UA remained normal. Chest/abdominal CTs were done 3 months and 9 months later with unremarkable findings. However, the patient complained of persistent progressively worsening malaise. Eleven months later, he was admitted again because of refractory anemia. At this time, bone marrow smear indicated erythroid hyperplasia and significant dyshaematopoiesis of all three lineages. The major loss of the D20S108 signal suggesting 20q- along with minor gains of D8Z2 signal was discovered by fluorescence in situ hybridization (FISH), which was consistent with a G banding karyotype. MDS was confirmed and the patient was classified as low-moderate risk group, hence no chemotherapy. He has been on regular clinic follow-up and the condition has not deteriorated.

### Discussion

The most commonly used diagnostic laboratory and clinical criteria for TLS are those proposed by Cairo and Bishop [3]. A clinical diagnosis of TLS includes one clinical symptom and two laboratory criteria, and at least two laboratory criteria must be present for three days before treatment or up to seven days after treatment. In our case, the patient fulfilled one clinical symptom (acute renal failure) and two laboratory criteria (hyperphosphatemia, hyperuricemia), thus meeting the clinical diagnostic criteria for TLS. In the absence of precipitating chemotherapy or radiotherapy, we considered this as a STLS case.

Acute TLS is usually observed after the initiation of cytotoxic chemotherapy of malignancies, especially hematological malignancies with a high turnover rate. The severity of TLS depends on tumor burden, tumor type, proliferation rate, baseline uric acid level and the sensitivity of the tumor to chemotherapeutic agents [4]. STLS prior to the initiation of chemotherapy is rare, especially in the absence of evidence of a tumor, including hematological malignancies and solid tumor. According to previous studies, STLS most commonly occurrs in Burkitt lymphoma and non T-cell acute lymphoblastic leukemia [2], but it has also been reported in acute lymphoblastic leukemia (ALL) [4], acute myeloid leukemia(AML) [5], myelofibrosis [6], metastatic germ cell tumor [7] and solid tumors [8].

To the best of our knowledge, our case is the first STLS reported in association with MDS. The initial presentations of significant hyperuricemia, hyperphosphatemia, metabolic acidosis, AKI and relative hypocalcemia were all consistent with STLS. In 2003, Yang et al. [9] reported a 32-year-old man who had been diagnosed as MDS with refractory anemia and excess blasts in transformation subtype developed TLS after a single 1.0 g dose of methylprednisolone. However, in our case, the patient did not fulfill the diagnostic criteria for MDS at the onset of STLS. Instead, he was diagnosed as MDS almost one year after the episode of STLS. When the STLS occurred, repeated bone marrow cytology and biopsy did not show evidence of a large tumor burden or of tumor types which had been reported in association with STLS. The etiology in our case is inconclusive. Potential causes for STLS include endogenous secretion of glucocorticoid with infection [10] and fever [8]. In Daisuke et al.’s case of STLS in ALL [4], it was believed the STLS might have been triggered by a febrile gastroenteritis-like illness one month prior to the first admission and accelerated by the concomitant urinary tract infection. In our case, the patient had experienced abdominal pain consistent with cholecystitis before the admission and it had been only one weeks after a surgery when the AKI developed. The endogenous secretion of glucocorticoid triggered by the prior infection and the stress of the operation could be considered to be causes of the STLS.

## Conclusions

We report an adult presenting with AKI and laboratory features of acute TLS months before his diagnosis of MDS, in the absence of precipitating chemotherapy and radiotherapy. It has been suggested occult malignancy should be considered in the case of unexplainable AKI with hyperuricemia [11]. Aggressive examination for malignancies, including solid tumors and hematological malignancies should be thoroughly performed and regularly repeated in this population, and patients without a definite diagnosis should be closely followed-up in the clinic.

### Consent

Written informed consent was obtained from the patient for publication of this Case report and any accompanying images. A copy of the written consent is available for review by the Editor of this journal.

## References

[1] Jasek AM, Day HJ (1994). Acute spontaneous tumor lysis syndrome. Am J Hematol.

[2] Hsu HH, Chen YC, Tian YC, Chan YL, Kuo MC, Tang CC, Fang JT, Lee SY, Yang CW (2009). Role of serum sodium in assessing hospital mortality in cancer patients with spontaneous tumor lysis syndrome inducing acute uric acid nephropathy. Int J Clin Pract.

[3] Cairo MS, Bishop M (2004). Tumour lysis syndrome: new therapeutic strategies and classification. Br J Haematol.

[4] Kobayashi D, Wofford M, McLean TW, Lin JJ (2010). Spontaneous tumor lysis syndromes in a child with T-cell acute lymphoblastic leukemia. Pediatr Blood Cancer.

[5] Riccio B, Mato A, Olson EM, Berns JS, Luger S (2006). Spontaneous tumor lysis syndrome in acute myeloid leukemia: two cases and a review of the literature. Cancer Biol Ther.

[6] Sile S, Wall BM (2001). Acute renal failure secondary to spontaneous acute tumor lysis syndrome in myelofibrosis. Am J Kidney Dis.

[7] Pentheroudakis G, O'Neill VJ, Vasey P, Kaye SB (2001). Spontaneous acute tumor lysis syndrome in patients with metasitatic germ cell tumors. Report of two cases. Support Care Cancer.

[8] Levin M, Cho S (1996). Acute tumor lysis syndrome in high grade lymphoblastic lymphoma after a prolonged episode of fever. Med Pediatr Oncol.

[9] Yang SS, Chau T, Dai MS, Lin SH (2003). Steroid-induced tumor lysis syndrome in a patient with preleukemia. Clin Nephropathy.

[10] Chen RL, Chuang SS (2009). Transient spontaneous remission after tumor lysis syndrome trigged by a severe pulmonary infection in an adolescent boy with acute lymphoblastic leukemia. J Pediatr Hemotol Oncol.

[11] Sharma SK, Malhotra P, Kumar M, Sharma A, Varma N, Singh S (2005). Spontaneous tumor lsis syndrome in acute lymphoblastic leukemia. J Assoc Physicians India.

[CR12] The pre-publication history for this paper can be accessed here:http://www.biomedcentral.com/1471-2369/15/164/prepub

